# Intrapleural Bortezomib for the Therapy of Myelomatous Pleural Effusion: A Case Report

**DOI:** 10.1155/2012/978479

**Published:** 2012-10-11

**Authors:** Magdalena Klanova, Pavel Klener, Marek Trneny, Jan Straub, Ivan Spicka

**Affiliations:** ^1^1st Department of Medicine and Clinical Department of Hematology, the General Teaching Hospital, Charles University in Prague, Prague, Czech Republic; ^2^Institute of Pathological Physiology, First Faculty of Medicine, Charles University in Prague, Prague, Czech Republic; ^3^Institute of Hematology and Blood Transfusion, Prague, Czech Republic

## Abstract

Myelomatous pleural effusion (MPE) is an extremely rare manifestation of multiple myeloma (MM). We present a case of MPE in a patient with IgG-*κ* MM treated with intrapleural bortezomib with systemic bortezomib-based therapy. Although we observed good local response, the patient succumbed due to systemic myeloma progression.

## 1. Introduction

Myelomatous pleural effusion is an extremely rare manifestation of multiple myeloma and only a few cases have been reported to date [1]. MPE in patients with MM is often associated with high-risk disease and poor prognosis despite aggressive treatment. There is no standard treatment strategy for MPE and the majority of cases of MM presenting with MPE were associated with resistance to therapy [2]. Bortezomib (the dipeptidylboronic acid analogue, proteasome inhibitor) belongs now to backbone antimyeloma drugs. The data concerning intrapleural administration of bortezomib is scarce. We present a case of MPE in a patient with IgG-*κ* MM treated with intrapleural bortezomib and concomitantly with systemic bortezomib-based-combined therapy. 

## 2. Case History

A 43-year-old female initially presented to the hospital in November 2009 with a dry cough, dyspnea, and left chest pain. The chest X-ray showed bilateral pneumonia. The computer tomography demonstrated a massive tumor involving the left breast and chest cavity, a tumor in the uterus and right ovary, multiple osteolytic lesions in the skull and ribs, and bilateral axillar lymphadenopathy. Immunohistochemical analysis of the breast tumor biopsy revealed plasmacytoma/multiple myeloma. Bone marrow examination by trephine biopsy revealed 30% infiltration with atypical plasma cells. Cytogenetic studies including FISH showed a deletion of chromosome 13 and amplification of 1q21 and 1q26 genomic regions. On admission the total plasma protein was 106 g/L, albumin 40 g/L, urea 5.7 mmol/L, creatinine 74 umol/L, calcium 2.12 mmol/L, LDH 3.7 ukat/L (normal values 2,2–3,75 ukat/L), and *β*-2 microglobulin 3.0 mg/L (normal values 1.0–2,4 mg/L). The international staging system (ISS) score was 1. Serum protein electrophoresis with immunofixation confirmed monoclonal gammopathy IgG-*κ* 44 g/L. Initially, the patient was treated with cyclophosphamide, thalidomide, and dexamethasone (CTD), but achieved no objective response (stable disease). In the second line therapy bortezomib (Velcade) was added (VTD combination) but due to side effects (severe myopathy) dexamethasone had to be shortly discontinued. 

**Figure 1:**
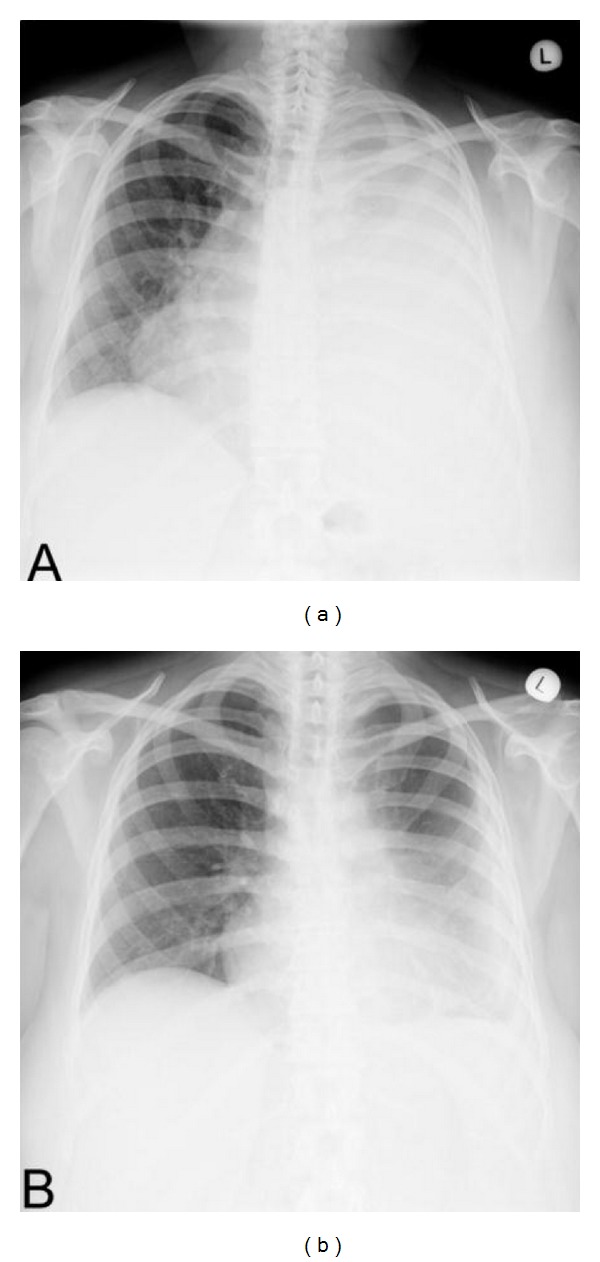
(a) Chest X-ray of patient showing massive left pleural effusion and (b) reduction of the pleural effusion after two doses of intrapleural bortezomib.

In July 2010, after 2 VT cycles, the CT scan showed a progression of extranodal masses with massive left pleural effusion (Figure 1(a)). The flow-cytometry analysis of pleural effusion demonstrated infiltration with atypical plasma cells thereby confirming the diagnosis of MPE. Repeated evacuations of the pleural fluid resulted only in short alleviations followed by rapid replenishment of the MPE. The patient was indicated to third-line therapy and received 1 modified cycle of bortezomib, doxorubicin, and dexamethasone (PAD), in which, on days 8 and 11, one-half of the bortezomib dose (i.e., 0.75 mg/m^2^) was administered intrapleurally and the other half (i.e., 0.75 mg/m^2^) intravenously. The patient thus received two doses of intrapleural bortezomib (0.75 mg/m^2^) three days apart, namely, in an attempt to mitigate the symptoms associated with pleural effusion refractory to repeated thoracocenteses. After the intrapleural administration of bortezomib the patient became thoracocentesis-independent, and in a two-week period the CT scan confirmed significant regression of pleural effusion (Figure 1(b)). Subsequently, the patient received four cycles of fourth-line therapy—PAD with lenalidomide (added to combination due to the progression of extramedullary disease) but still with no effect on extramedullary tumors. Due to the progression of the disease and poor tolerance of previous therapy (hypotension after bortezomib, cytopenia) immediate high-dose melphalan therapy with stem cell support was planned; however, the patient eventually succumbed in November 2010 due to infectious complications just before the start of stem cell mobilization. The autopsy was not performed at the request of the family. 

## 3. Discussion

Multiple myeloma is the second most common hematologic malignancy after non-Hodgkin's lymphoma and is responsible for 2% of cancer deaths [3]. The plasma cell disorder is characterized by the proliferation of malignant plasmocytes accumulating mainly in the bone marrow and by the production of monoclonal immunoglobulin [4]. Apart from this characteristic features the clinical manifestations of disease could be variable. Pleural effusion is an untypical finding in myeloma patients affecting approximately 6% of the cases [1]. The pathogenesis most frequently involves congestive heart failure due to amyloidosis and cardiac disease in the older myeloma population [5, 6]. MPE itself due to involvement of the pleura is extremely rare and only a few cases have been reported to date. Kintzer et al. reported 0.8% cases of MPE from 958 patients with MM [1]. MPE usually occurs as a late complication of MM in the course of the disease progression and is associated with very poor prognosis. A survival rate of less than 4 months has been reported in the few cases of MPE despite aggressive treatment [6]. 

There is no standard therapy for MPE. Various anti-myeloma agents in combination with local treatment approaches targeted at pleural effusion, for example, pleurodesis, were tested without significant effect. High-dose chemotherapy with peripheral blood stem cell support for MPE did not confer clear survival advantage [7]. The intrapleural administration of *α*-interferon [8] or doxorubicin [9] has been tested in an attempt to increase concentrations of the drugs in the pleural cavity. The data concerning intrapleural administration of bortezomib remains scarce. Iannitto et al. published a case report of intrapleurally administered bortezomib in a patient with refractory MM, in whom MPE occurred late in the course of the disease. Their patient first received two cycles of i.v. bortezomib, dexamethasone, and pegylated liposomal doxorubicin without any impact on the formation of pleural effusion. Thus, the therapy was modified and in the third cycle half of the bortezomib dose was administered intrapleurally (for the total of 4 injections). After completion of the single-modified cycle of bortezomib, MPE disappeared [10]. Similarly, in our patient only as few as two intrapleural administrations of bortezomib (0.75 mg/m^2^ each) resulted in a rapid improvement of clinical symptoms, followed by a gradual disappearance of MPE. We assume that increased intrapleural concentration of bortezomib as a result of local administration might represent a major factor responsible for the rapid remission of MPE. In conclusion, intrapleural administration of bortezomib appears safe and efficacious treatment approach targeted at MPE and MPE-related clinical symptoms. 

## References

[1] Kintzer JS, Rosenow EC, Kyle RA (1978). Thoracic and pulmonary abnormalities in multiple myeloma. A review of 958 cases. *Archives of Internal Medicine*.

[2] Kim YM, Lee KK, Oh HS (2000). Myelomatous effusion with poor response to chemotherapy. *Journal of Korean Medical Science*.

[3] Kyle RA, Rajkumar SV (2008). Multiple myeloma. *Blood*.

[4] Bataille R, Harousseau JL (1997). Multiple myeloma. *New England Journal of Medicine*.

[5] Rodriguez JN, Pereira A, Martinez JC, Conde J, Pujol E (1994). Pleural effusion in multiple myeloma. *Chest*.

[6] Hughes JC, Votaw ML (1979). Pleural effusion in multiple myeloma. *Cancer*.

[7] Kamble R, Wilson CS, Fassas A (2005). Malignant pleural effusion of multiple myeloma: prognostic factors and outcome. *Leukemia and Lymphoma*.

[8] Makino S, Yamahara S, Nagake Y, Kamura J (1992). Bence-Jones myeloma with pleural effusion: response to α-interferon and combined chemotherapy. *Internal Medicine*.

[9] Iannitto E, Scaglione R, Musso M, Abbadessa V, Licata G (1988). Intrapleural adriamycin in treatment of myelomatous pleural effusion: a case report. *Haematologica*.

[10] Iannitto E, Minardi V, Tripodo C (2007). Use of intrapleural bortezomib in myelomatous pleural effusion. *British Journal of Haematology*.

